# Resolution of Hypothyroidism Restores Cold-Induced Thermogenesis in Humans

**DOI:** 10.1089/thy.2018.0436

**Published:** 2019-04-09

**Authors:** Claudia Irene Maushart, Rahel Loeliger, Gani Gashi, Mirjam Christ-Crain, Matthias Johannes Betz

**Affiliations:** Department of Endocrinology, Diabetes and Metabolism, University Hospital Basel and University of Basel, Basel, Switzerland.

**Keywords:** thyroid, hypothyroidism, thyroid hormone, cold-induced thermogenesis, energy expenditure, brown adipose tissue

## Abstract

***Background:*** Hypothyroidism is a frequent endocrine disorder with common symptoms of increased cold sensitivity and unintended weight gain, indicating changes in energy expenditure (EE) and response to cold exposure. Thyroid hormones (TH) play an important role for proper function of brown adipose tissue (BAT) and cold-induced thermogenesis (CIT) in rodents, but the role of hypothyroidism on CIT in humans is uncertain.

***Methods:*** This was a prospective observational study. Forty-two patients presenting with subclinical or overt hypothyroidism in whom TH replacement was planned were recruited. Thirty-three patients completed the study. Thermogenesis was measured by indirect calorimetry during warm conditions and after a mild cold stimulus of 90 minutes, both during the hypothyroid state and after at least three months of sufficient TH replacement. CIT was determined as the difference between EE during mildly cold and warm conditions. The primary endpoint was the change of CIT between the hypothyroid and euthyroid state.

***Results:*** EE during warm conditions increased from a median of 1330 (interquartile range [IQR] 1251–1433) kcal/24 hours in the hypothyroid state to a median of 1442 (IQR 1294–1579) kcal/24 hours in the euthyroid state (+8.5%; *p* = 0.0002). EE during mild cold exposure increased from 1399 (IQR 1346–1571) kcal/24 hours to 1610 (IQR 1455–1674) kcal/24 hours (+15%; *p* < 0.0001). The median CIT was 55 (IQR 1–128) kcal/24 hours at the baseline visit, after restoration of euthyroidism CIT increased by 102% to a median of 111 (IQR 15.5–200) kcal/24 hours (*p* = 0.011). Serum levels of free thyroxine at the respective visit and mean outdoor temperature during the preceeding 30 days were significantly associated with CIT (*p* = 0.021 and *p* = 0.001, respectively).

***Conclusion:*** Restoring euthyroidism significantly increases CIT in hypothyroid humans.

## Introduction

Hypothyroidism is a frequent endocrine disorder, with the prevalence rate of mild or subclinical hypothyroidism being 4.3% and of overt hypothyroidism being 0.3%. Patients frequently complain of increased cold sensitivity and involuntary weight gain, indicating changes in energy expenditure (EE) and response to cold exposure (1).

Thyroid hormone (TH) plays an important role in human energy homeostasis. The basal metabolic rate (BMR), which is the main component of human EE, is well known to be regulated by TH and to be reduced in hypothyroidism (2–4). Multiple cellular pathways and mechanisms are influenced by TH via TH receptors, which are ligand-activated transcription factors belonging to the family of nuclear hormone receptors. TH increases the production of adenosine triphosphate (ATP) and at the same time stimulates futile cycles involving Na+/K+ATPase and the sarcoplasmatic/endoplasmatic reticulum Ca^2+^ATPase (SERCA) in muscle, which leads to increased ATP consumption. It also increases the leak of protons across the inner mitochondrial membrane, leading to less efficient oxidative phosphorylation and more heat release. Additionally, TH plays an important role in the development and function of brown adipose tissue (BAT), which is a thermogenic tissue (5). Brown adipocytes are densely packed with mitochondria, which contain uncoupling protein 1 (UCP1) in the inner mitochondrial membrane, which is exclusive to these cells and can convert chemical energy directly into heat by short-circuiting the respiratory chain (6). The importance of TH for BAT is illustrated by the fact that in response to cold brown adipocytes express high levels of deiodinase 2 (DIO2), which converts thyroxine (T4) to the more active triiodothyronine (T3) (7,8). The high local levels of T3 are pivotal for both mitochondriogenesis (9) and expression of UCP1 (10).

In humans and other mammals, core body temperature is closely regulated, and several autonomous mechanisms exist to preserve it. BMR and EE from physical activity are the main contributors to temperature homeostasis in humans. Additionally, in response to mild cold exposure, the body can reduce heat loss by cutaneous vasoconstriction (11). If these mechanisms are insufficient to maintain core temperature, EE can be increased by non-shivering thermogenesis in muscle or BAT, which is called “adaptive thermogenesis” or “cold-induced thermogenesis” (CIT) (12).

Data from animal experiments indicate that severe hypothyroidism is associated with drastically reduced CIT. In these experiments, hypothyroidism was induced by high doses of propylthiouracil (13), which also blocks peripheral deiodination, or animals were severely hypothyroid and exposed to very low temperatures (14). This is comparable to severe lack of TH in humans, which can lead to myxedema coma and is associated with hypothermia. The effect of mild to moderate hypothyroidism on CIT in humans is less clear, and only limited data from small prospective studies are available (15,16). Theoretically, a lower BMR as a consequence of hypothyroidism could lead to increased CIT in order to compensate for the lower contribution of BMR to heat production. On the other hand, lack of TH could reduce CIT and lead to greater reliance on vasoconstriction and insulation due to the same molecular effects that affect BMR in hypothyroidism. This study therefore sought to investigate whether hypothyroidism reduces CIT compared to the euthyroid state.

## Methods

### Subjects

A prospective observational study was conducted in patients with hypothyroidism. Between April 2015 and April 2017, 42 patients were enrolled, aged between 18 and 65 years, presenting to the outpatient endocrine clinic at the University Hospital Basel with subclinical or overt hypothyroidism defined as thyrotropin (TSH) level >4.5 mIU/L or a free T4 (fT4) level <10 pmol/L, and in whom TH replacement was clinically indicated.

Patients with a body mass index (BMI) >30 kg/m^2^, uncontrolled diabetes, treatment with glucocorticoids, pregnancy or breastfeeding, known hypersensitivity to cold (e.g., primary or secondary Raynaud's syndrome), abuse of alcohol or illicit drugs, or any other significant chronic or acute disease such as heart or kidney failure or liver cirrhosis were excluded.

All participants provided written informed consent. The study protocol was reviewed and approved by the medical ethics committee of the University of Basel (ID EKNZ 2015-028) and registered on ClinicalTrials.gov (NCT02364102).

Levothyroxine treatment was started as indicated by the treating physician and patients were seen for the first study visit within the first two weeks after screening.

### Measurements of EE and CIT

At each visit, EE was measured during warm conditions (EE_warm_) and after mild cold exposure (EE_cold_) by indirect calorimetry for 30 minutes using a ventilated hood calorimeter (Cosmed Quark RMR; Cosmed, Rome, Italy). All measurements took place in an air-conditioned study room at a controlled ambient temperature of 24°C year round. For determination of EE_warm_, participants were placed in a hospital bed in a supine position and were covered with a fleece blanket. After the first measurement, the blanket was removed, and the patients were asked to remove all clothes except for a t-shirt and shorts. Then we induced mild cold exposure using a water circulated cooling system (Hilotherm clinic; Hilotherm GmbH, Argenbühl, Germany) around the patient's midsection. The water temperature was continuously reduced by 1°C every two minutes from 25°C to a minimum of 14°C. During the cooling, the participants were observed for signs of shivering and regularly asked if they experienced shivering. In case of shivering, they were covered with a blanket for five minutes, and the water temperature was raised by 2°C until the shivering stopped. The total cooling time was 90 minutes. During the last 30 minutes of cooling, the second measurement of EE_cold_ was performed.

Additionally, all patients were repetitively asked to rate their cold sensations on a visual analog scale (VAS; ranging from 1 = “cold” to 7 = “hot”). The core body temperature was measured with infrared tympanometry (ThermoScan PRO 6000; Braun, Marlborough, MA) before and after cold exposure.

**FIG. 1.**
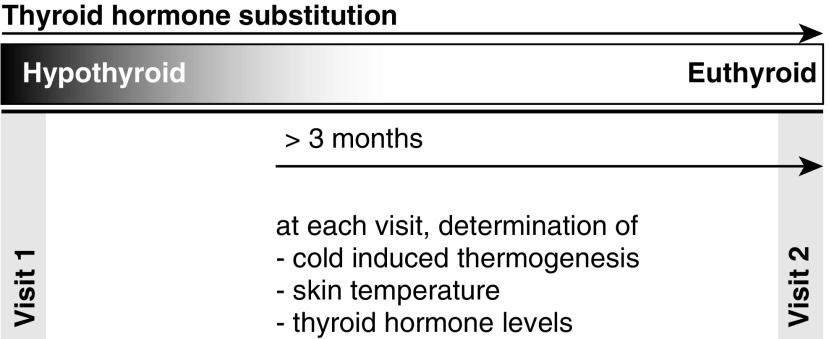
Chart of study schedule.

These measurements were carried out first in the hypothyroid state (visit 1) and after at least three months of sufficient TH replacement (visit 2). CIT was calculated as the difference between EE_cold_ and EE_warm_ (Fig. 1).

### Laboratory analysis

At both visits, serum TSH, free T3 (fT3), fT4, and a general laboratory analysis including a lipid profile were performed in all patients. All routine analyses were conducted at the central laboratory of the University Hospital Basel. For TSH and fT3/fT4, electro-chemiluminescence immunoassays (Elecsys; all assays from Roche Diagnostics AG, Rotkreuz, Switzerland) were used. The normal range for TSH was 0.332–4.490 mIU/L. The fT3 and fT4 levels had a reference range of 2.6–5.6 pmol/L for fT3 and 11.6–22.0 pmol/L for fT4, respectively.

### Measurement of body composition

Body composition was determined at each study visit using a four-electrode body impedance analysis device (Akern BIA 101; Akern SRL, Florence, Italy).

### Measurement of skin temperature

The skin temperature was measured continuously at seven defined body locations by wireless iButtons (Maxim Integrated, San Jose, CA). After the first 17 patients, four peripheral body locations were added for the remaining 19 participants. The locations were as follows: supraclavicular region (left and right), parasternal at the level of the second intercostal space (left and right), umbilicus, mid thigh (left and right), middle of the lower arm palmar side, finger tip of the third finger of the non-dominant hand, middle of the lower leg (left), and back of the left foot (17).

### Meteorological data

Outdoor temperatures were recorded by the Institute for Meteorology, Climatology, and Remote Sensing at the University of Basel at an urban meteorological station in close vicinity of the University Hospital. Daily mean, maximum, and minimum temperatures were provided for all days during the study period. Mean daily temperatures were averaged over a period of 30 days prior to the respective study visit using a sliding average function.

### Statistical analysis

It was speculated that the difference in CIT would be in the order of magnitude of 20% in patients with hypothyroidism. Therefore, to achieve a power of 80% at an alpha of 5%, a minimum of 33 patients needed to be examined. In order to account for dropout of participants, the aim was to include 40 participants in the study. The primary endpoint was analyzed by Wilcoxon signed-rank test. Data analysis was performed using GraphPad Prism v7 (GraphPad Software, La Jolla, CA). In order to assess the effects of TH levels on CIT at both study visits (repeated measures), a linear mixed-effects model with a random intercept was fitted to the data with the R software package v3.4.1 (18) using the nlme package (19). CIT was inserted into the formula as response variable, and fT4 and outdoor temperature as explanatory variables associated with fixed effects. The data were grouped by the individuals' study ID as a random effect (random intercept).

A *p*-value <0.05 was considered significant. Data are given as the median with interquartile range (IQR) unless stated otherwise.

## Results

### Baseline patient data and hormone levels

Forty-two patients were recruited to the study. Of these, three were lost to follow-up, two did not attend the second study visit because of a lack of time, one was excluded due to pregnancy, and stable euthyroid hormone levels could not be attained in three patients due to a lack of compliance. The clinical characteristics of the remaining 33 patients who completed the study are summarized in Table 1.

**Table 1.** Clinical Data

	*Baseline*	*Follow-up*	p
Age	44 (24.5–56)		
Sex (% female)	78.8		
BMI	25.3 (23.4–26.5)	25.0 (23.4–27.2)	0.25
TSH at screening (mIU/L)	17.0 (10.8–53.0)		
fT4 at screening (pmol/L)	10.8 (5.7–14.9)		
TSH baseline (mIU/L)	10.88 (6.8–40.1)	1.73 (0.3–3.9)	<0.0001
fT4 (pmol/L)	12.2 (9.7–17.2)	19 (17.0–21.2)	<0.0001
fT3 (pmol/L)	4.1 (3.3–4.6)	4.4 (4.0–5.0)	0.068
Body muscle mass %	53.5 (48–59.8)	53 (48.5–59)	0.32
Body fat mass %	25.5 (15.3–32.3)	23 (15–28.5)	0.38
Estimated BMR (kcal/day)	1365 (1300–1527)	1392 (1297–1585)	0.46
Glucose (mmol/L)	4.8 (4.5–4.9)	4.8 (4.4–5.1)	0.94
Triglycerides (mmol/L)	1.0 (0.7–1.23)	1.0 (0.8–1.4)	0.70
LDL cholesterol (mmol/L)	3.1 (2.2–3.4)	2.6 (2.1–3.3)	0.063
Total cholesterol (mmol/L)	4.9 (4.4–5.6)	4.7 (4.0–5.3)	0.023
HDL cholesterol (mmol/L)	1.8 (1.5–2.1)	1.6 (1.3–1.8)	0.0002

Data are given as the median and interquartile range (*n* = 33).

BMI, body mass index; TSH, thyrotropin; fT4, free thyroxine; fT3, free triiodothyronine; BMR, basal metabolic rate; LDL, low-density lipoprotein; HDL, high-density lipoprotein.

At screening, all patients were hypothyroid with a TSH level >4.5 mIU/L or a fT4 level <10 pmol/l. At the time of the first visit, five of the included patients were already back in the normal TSH reference range, as TH substitution had already been started for several days. One of the patients was suffering from secondary hypothyroidism, and TSH was not recorded. After starting TH replacement therapy, regular laboratory controls of TH values were scheduled, as indicated by the treating physician. The following concomittant medications were recorded (number of patients taking medication): cardioselective betablockers (*n* = 3), ACE inhibitors (*n* = 2), statins (*n* = 3), hormonal contraception (*n* = 9), and thyrostatic drugs (*n* = 2).

The second study visit took place at least three months after patients had attained normal values for TSH and fT4. The median time between the first and second study visit was 160 days (IQR 128–225 days). At the second visit, levels of TSH were significantly lower and levels of fT4 significantly higher. BMI and body composition did not change. Levels of total cholesterol, low-density lipoprotein cholesterol, and high-density lipoprotein cholesterol decreased from baseline to second visit, while triglyceride and glucose levels remained stable (Table 1).

### Skin temperature and cold sensation

In order to assess the insulating response to the cold stimulus, skin temperature was measured at predefined positions. Surface temperatures dropped at all measured locations, with the exception of the supraclavicular area. Core temperature remained stable (Table 2). A trend for higher supraclavicular skin temperatures was observed during the second study visit.

**Table 2.** Temperatures and Cold Sensation

*Location*	*Hypothyroid warm*	*Hypothyroid cold*	*Euthyroid warm*	*Euthyroid cold*	p *for hypo- vs. euthyroid*	
					*Warm*	*Cold*
*Skin temperature (in °C)*						
Supraclavicular	35.14 ± 0.44	35.2 ± 0.48	35.36 ± 0.70	35.40 ± 0.65	0.081	0.064
Parasternal	34.68 ± 0.62	34.22 ± 0.92^**^	34.57 ± 1.11	34.43 ± 0.62	0.86	0.24
Thigh	32.62 ± 0.62	31.11 ± 0.92^***^	32.35 ± 1.51	31.11 ± 1.01^***^	0.34	0.97
Arm	34.19 ± 1.33	29.1 ± 1.05^***^	34.07 ± 1.51	29.54 ± 2.03^***^	0.99	0.40
Finger	34.22 ± 2.01	26.09 ± 2.08^***^	34.5 ± 2.05	26.26 ± 2.33^***^	0.55	0.98
Leg	32.45 ± 1.47	30.39 ± 0.68^***^	32.57 ± 1.25	30.49 ± 1.38^***^	0.72	0.90
Foot	31.72 ± 2.03	28.64 ± 1.35^***^	32.55 ± 2.25	29.39 ± 1.62^***^	0.23	0.02
*Core temperature (in °C)*						
	36.79 ± 0.28	36.91 ± 0.33	36.73 ± 0.26	36.72 ± 0.44	0.58	0.90
*Cold sensation (VAS)*						
	4.6 ± 0.8	1.5 ± 0.6^***^	5.0 ± 1.1	1.5 ± 0.7^***^	0.076	0.68
*Water temperature at the end of cooling (in °C)*						
		16.3 ± 1.3		15.8 ± 1.3		0.13

Data shown are the mean ± standard deviation. Asterisks indicate significant changes of skin temperatures and cold sensation after cooling for all measured locations within the same study visit (^**^p = 0.01; ^***^p < 0.001).

VAS cold sensation scores: 7 = “hot,” 4 = “neutral,” 1 = “cold.”

VAS, visual analog scale.

All participants were intermittently asked about their subjective cold sensation, which was documented at baseline and after the cooling period. The water within the cooling system reached a mean temperature of 16°C at the end of the cooling period. The drop in skin temperature and the subjective cold sensation corresponded well, which clearly indicated that an adequate cooling protocol was used.

### Resting EE at warm and cold temperatures

**FIG. 2.**
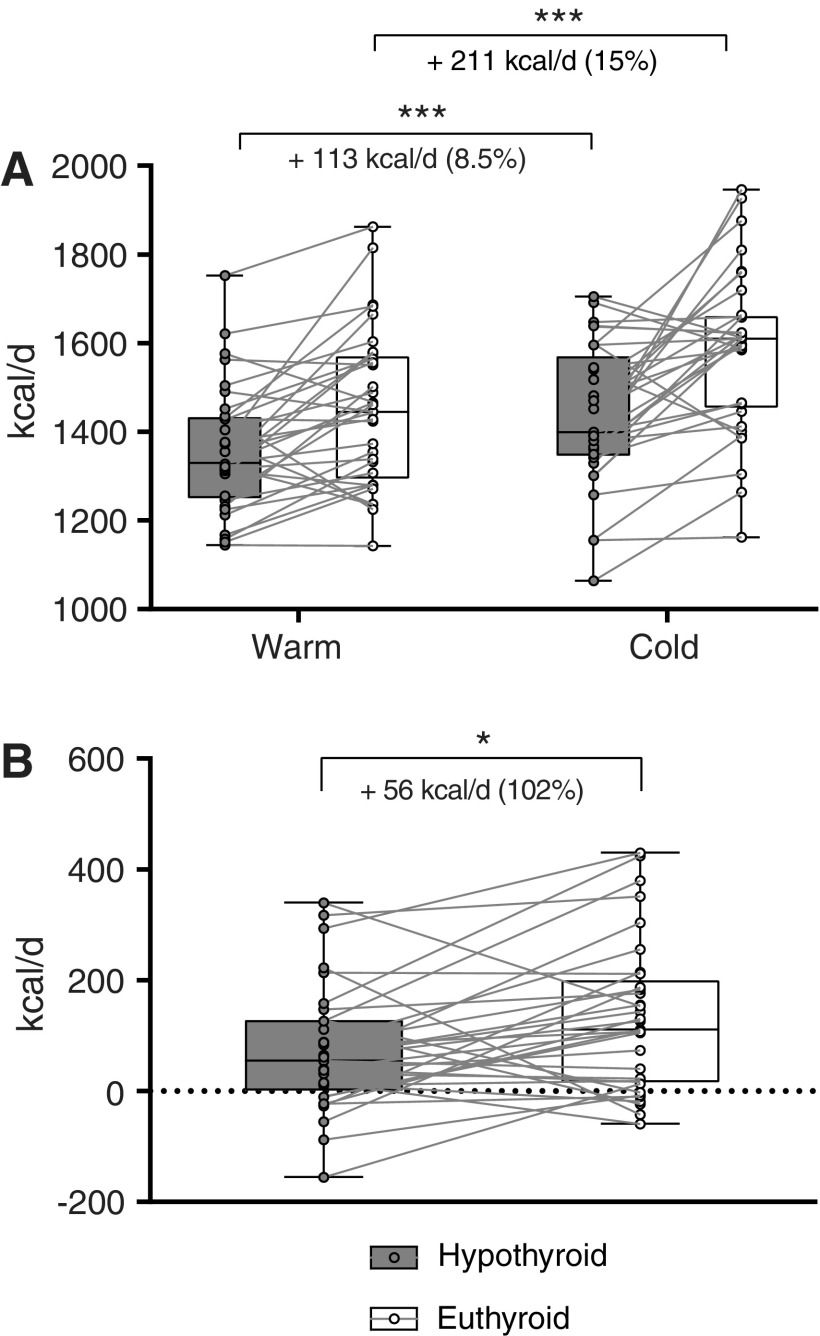
(**A**) The median energy expenditure (EE) increased from the hypothyroid to the euthyroid condition at warm temperatures by 113 kcal/day (8.5%; *p* = 0.0002) and after cooling by 211 kcal/day (15%; *p* < 0.0001). (**B**) Cold-induced thermogenesis (CIT) rose by 56 kcal/day (102%; *p* = 0.011) after thyroid hormone replacement compared to the hypothyroid state. ***p < 0.001; *p < 0.05.

The median EE at warm temperature (EE_warm_) was 1330 (IQR 1251–1433) kcal/24 hours at the first visit (hypothyroid) and 1443 (IQR 1294–1579) kcal/24 hours at the second visit (euthyroid)—an increase of 8.5% (*p* = 0.0002). After the mild cold stimulus, EE was higher than during warm conditions at both visits. The median EE after the mild cold stimulus (EE_cold_) was 1399 (IQR 1346–1571) kcal/24 hours at the first visit and 1610 (IQR 1455–1674) kcal/24 hours at the second visit—an increase of 15% compared to the first visit (*p* < 0.0001; Fig. 2A).

### CIT before and after restoration of euthyroidism

The median CIT was 55 (IQR 1–128) kcal/24 hours at the first visit. CIT increased by 102% to a median CIT of 111 (IQR 15.5–200) kcal/24 hours (*p* = 0.011) at the second visit (Fig. 2B).

### Influence of TH status on EE

**FIG. 3.**
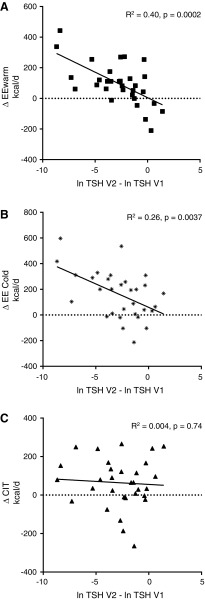
Relation between change in thyroid hormone status calculated as difference of log-converted thyrotropin levels at the two visits and change in energy expenditure: (**A**) ΔEE_warm_: *R*^2^ = 0. 40, *p* = 0.0002; (**B**) ΔEE_cold_: *R*^2^ = 0.26, *p* = 0.0037; and (**C**) ΔCIT: *R*^2^ = 0.004, *p* = 0.74. V1, visit 1 (hypothyroid); V2, visit 2 (euthyroid).

In order to estimate the effect of TH status on EE, linear regression was performed of the change in EE_warm_, EE_cold_, and CIT versus the change in TSH from visit 1 to visit 2. TSH values were logarithmically transformed in order to normalize their distribution. The change in TSH (ΔTSH) was significantly correlated to ΔEE_warm_ (*R*^2^ = 0.40, *p* = 0.0002) and ΔEE_cold_ (*R*^2^ = 0.26, *p* = 0.0037). However, it was not significantly correlated to ΔCIT (*R*^2^ = 0.004, *p* = 0.74; Fig. 3). Taking into account that at visit 1 the TSH of five patients was already back in the reference range, the same analysis was performed with the TSH values obtained at screening. The results for ΔEE_warm_, ΔEE_cold_, and ΔCIT were *R*^2^ = 0.47, *p* < 0.0001, *R*^2^ = 0.33, *p* = 0.0009, and *R*^2^ = 0.010, *p* = 0.28, respectively.

Similarly, the change in fT4 and fT3 levels was associated with the change in ΔEE_warm_ (*R*^2^ = 0.20, *p* = 0.009 and *R*^2^ = 0.28, *p* = 0.0014, respectively) and ΔEE_cold_ (*R*^2^ = 0.12, *p* = 0.049 and *R*^2^ = 0.21, *p* = 0.0071, respectively), but not with ΔCIT (*R*^2^ = 0.001, *p* = 0.83 and *R*^2^ = 0.01, *p* = 0.55, respectively; Supplementary Figs. S1 and S2).

### Influence of outdoor temperature on EE and CIT

**FIG. 4.**
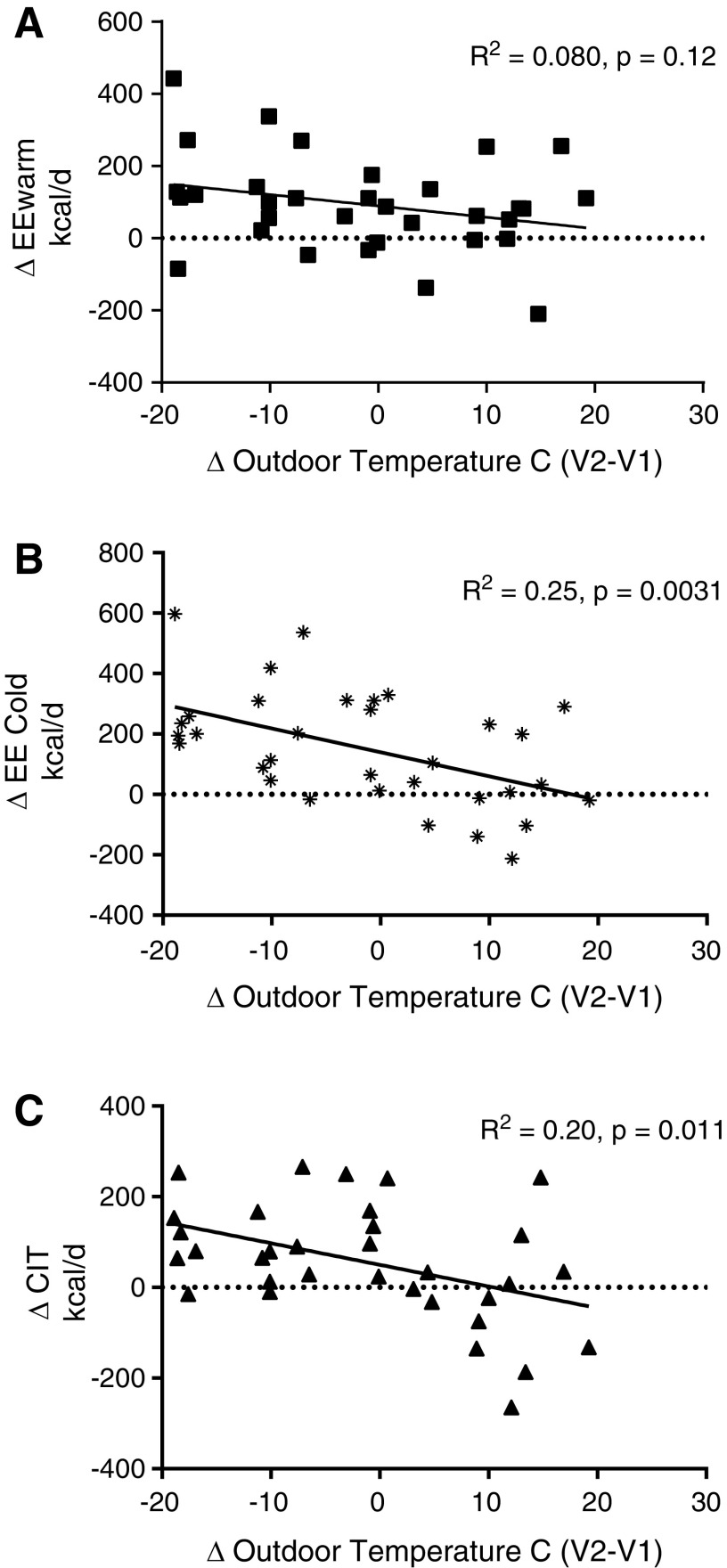
Relation between change in outdoor temperature and change energy expenditure: (**A**) ΔEE_warm_: *R*^2^ = 0.080, *p* = 0.12; (**B**) ΔEE_cold_: *R*^2^ = 0.25, *p* = 0.0031; and (**C**) ΔCIT: *R*^2^ = 0.19, *p* = 0.011.

**FIG. 5.**
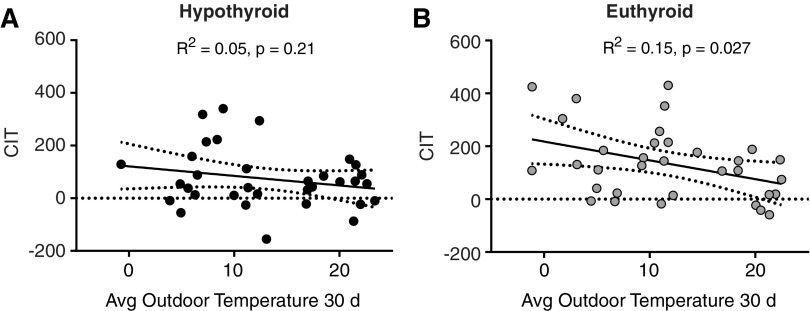
Influence of average outdoor temperature (°C) 30 days prior to the study visit on CIT (kcal/24 hours) in (**A**) hypothyroid and (**B**) euthyroid state.

Ambient temperatures and season affect CIT and BAT activity (20,21). Therefore, the daily outdoor temperatures were obtained during the whole study period, and the mean temperature during the 30 days preceeding the respective visit was calculated. The difference in mean outdoor temperature (ΔTemp) between the two visits with the change in EE_warm_, EE_cold_, and CIT was compared by linear regression. While ΔEE_warm_ was only weakly related to the difference in outdoor temperature (*R*^2^ = 0.078, *p* = 0.12), ΔEE_cold_ (*R*^2^ = 0.25, *p* = 0.0031) and ΔCIT (*R*^2^ = 0.19, *p* = 0.011) were significantly associated with ΔTemp (Fig. 4). Next, we assessed whether the mean outdoor temperature during the 30 days preceeding the respective visit was associated with CIT. At the hypothyroid visit, outdoor temperature during the 30 days prior to the visit was not significantly associated with CIT (*R*^2^ = 0.05, *p* = 0.21; Fig. 5A), while it was at the euthyroid study visit (*R*^2^ = 0.15, *p* = 0.027; Fig. 5B).

### Combined effect of outdoor temperature and TH status

In order to analyze the effects of outdoor temperature and TH status on CIT further and to avoid using differences between the values measured at both visits, a mixed-effects model was employed with CIT as the dependent variable. fT4 and mean outdoor temperature during the 30 days preceeding the respective visit as fixed effects and the respective subject as the random-effect variable were entered into the model. The estimated value was 6.02 ± 2.46 (*p* = 0.021) for fT4 and −6.01 ± 1.65 (*p* = 0.001) for the mean 30-day temperature (TEMP30D; see Table 3). The actual values and the values fitted to the model correlated well (*R*^2^ = 0.76). No significant statistical interaction between fT4 and TEMP30D was detected in the model.

**Table 3.** Results of Linear Mixed-Effects Model

*Random effects: ∼1|ID*					
	*(Intercept)*	*Residual*			
*SD*	79.94	81.53			

*Fixed effects: CIT ∼ fT4 + TEMP30D*					
	*Value*	*Std. Error*	*DF*	t*-Value*	p*-Value*
(Intercept)	80.54	46.07	32	1.75	0.090
fT4	6.02	2.46	31	2.44	0.021
TEMP30D	–6.01	1.65	31	–3.64	0.001

In order to assess the effects of thyroid hormone levels on CIT at both study visits (repeated measures), a linear mixed-effects model with a random intercept was fitted to the data. CIT was inserted as the response variable. fT4 and TEMP30D (outdoor temperature during the 30 days preceding the visit) were entered as fixed-effect variables. The individual study participant was inserted as a random-effect variable (random intercept).

CIT, cold-induced thermogenesis.

## Discussion

This study investigated the effect of restoring a euthyroid hormone status in patients with hypothyroidism on CIT. It demonstrates that CIT is reduced in spontaneously hypothyroid patients and that substitution of T4 in a dose to restore euthyroid TH levels significantly increases CIT.

Previously, two small prospective studies addressed this issue in patients with thyroid carcinomas who were undergoing TH withdrawal prior to therapy with radioactive iodine (RAI) (15,16). One study included 10 patients after approximately seven weeks of complete TH withdrawal who were clearly hypothyroid with a mean TSH level of 107 mIU/L and a mean fT4 level of 6 pmol/L. After four months of TH replacement, patients were mildly hyperthyroid. Both CIT and BAT activity increased significantly compared to the hypothyroid state (15). In the second study, six patients were studied after four weeks of treatment with T3 and subsequent complete TH withdrawal of two weeks and several months later in a thyrotoxic state. Mean CIT decreased from approximately 198 kcal/day to 143 kcal/day, and BAT activity as assessed by ^18^F-labeled fluoro-2-deoxyglucose (^18^F-FDG) positron emission tomography/computed tomography (PET/CT) did not change significantly. T3 can directly activate UCP1, even without a sympathetic stimulus (22), and hyperthyroidism has been demonstrated to increase BAT activity in humans (23). Notably, in both studies, CIT and BAT activity were measured in a hyperthyroid and not in a euthyroid state at the second study visit, which might have affected the results and may therefore preclude conclusions drawn on CIT in the hypothyroid state. This study therefore investigated a larger group of patients and aimed to achieve TSH levels within the reference range at the second study visit.

Impaired TH signaling has paradoxically been shown to increase BAT activity and CIT in mice through impaired vasoconstriction and thus increased heat loss (24). This study therefore measured skin temperatures at both peripheral and central skin positions. With the exception of the foot, no significant differences in skin temperature were detected between the two visits, indicating that hypothyroidism did not significantly impair cutaneous vasoconstriction in the present cohort.

As expected, the change in TH status correlated well to the change in EE_warm_, which is a close estimate of BMR. Surprisingly, a significant influence of TH status on CIT was not found when performing univariate linear regression analysis, which is in accordance with previous findings (15). Therefore, an attempt was made to analyze this apparent contradiction further.

CIT is very important for mammals exposed to cold, and research in rodents clearly demonstrates that adaptive thermogenesis can persist even if an important pathway fails (25). In line with this notion, significant CIT was also observed in patients who were severely hypothyroid (i.e., TSH >100 mIU/L), which is in agreement with the findings in patients undergoing RAI therapy after five weeks of complete TH withdrawal (15). The highly elevated TSH levels might directly stimulate BAT, as the TSH receptor is expressed in brown adipocytes and is coupled to G_s_ and the cyclic adenosine monophosphate signaling cascade (26), which increases lipolysis and activates UCP1. Indeed, hypothyroid mice with an inactivating mutation of the TSH receptor suffered from hypothermia when exposed to cold. *In vivo* transfection of brown adipocytes with wild-type TSH receptor rescued CIT in these hypothyroid animals (27). Moreover, hypothyroidism induces the expression of DIO2 (28), which regulates the local availability of T3 in BAT. Through this mechanism, high levels of TSH (29) might counteract the effects of hypothyroidism by maintaining normal T3 levels (30). In the present cohort, levels of fT3 were only slightly higher at the euthyroid study visit than during the hypothyroid visit. This may be due to the fact that the study mainly included patients with moderate hypothyroidism in whom levels of T3 often remain within the reference range (31).

TH sensitizes tissues to the action of catecholamines (13), which is important, as cold adaptation is driven by norepinephrine secretion from the sympathetic nervous system (32). Thus, it has been demonstated in rodents that TH is crucial for adaptation to a cold environment (33). However, if animals are rendered hypothyroid after cold adaptation, CIT and BAT function are virtually unchanged (34). In healthy humans, CIT is significantly influenced by seasonal changes in outdoor temperatures, which can lead to a complete suppression of CIT in summer (35,36). During chronic or repetitive cold exposure, the sympathetic nervous system secretes norepinephrine in BAT depots, which acutely activates thermogenesis (32). Moreover, adrenergic stimulation is crucial for the transdifferentiation of white adipocytes into brown adipocytes (37) (also called “brite”—brown in white adipocytes), which can be reversed by exposure to warm temperatures and the subsequently lower local levels of norepinephrine (38). In order to compensate for the influence of outdoor temperatures, the average temperatures of the month preceding the respective visit were included into a mixed-effects model. A significant influence of both outdoor temperature and fT4 was then observed on CIT. Importantly, fT4 and the outdoor temperature together account for approximately 75% of the variation of CIT.

Importantly, an inverse association was observed between outdoor temperatures during the month prior to the measurement and CIT only in the euthyroid state. We would therefore like to speculate that hypothyroidism limits the adaptation to environmental cold exposure in humans (Fig. 5).

Our study has several limitations. Most importantly, BAT function was not assessed by ^18^F-FDG-PET/CT, and thus it cannot be excluded that muscle contributed to CIT by non-shivering mechanisms such as calcium cycling. FDG-PET directly visualizes BAT function and would have allowed whether BAT depots expand or just increase their metabolic activity to be investigated. However, PET/CT scanning is associated with a non-negligible exposure to ionizing radiation, which would have posed ethical difficulties in an observational study with otherwise healthy and primarily young female patients. At least, it would have impeded recruitment for the study. It is not surprising that the two previous trials in humans were performed in patients undergoing RAI treatment in comparison to which the radiation dose from PET/CT is insignificant. Furthermore, several patients had already received T4 substitution between the screening date and the first visit. Therefore, the effect of TH substitution on CIT might have been underestimated. However, EE data were re-analyzed with the screening lab, which yielded comparable results.

A strength of our study is its relatively large size and heterogenous patient population, representing the real-world situation in the endocrine clinic. TH substitution was aimed at reaching euthyroid values. Thus, the detected differences in EE and CIT are very likely due to hypothyroidism at the first visit and not due to mild hyperthyroidism at the second visit.

Taken together, this study shows that even moderate levels of hypothyroidism reduce CIT in humans and that sufficient replacement of TH restores CIT. This effect might be due to reduced recruitment of brown adipocytes during the cold season and warrants further investigation.

## Supplementary Material

Supplemental data

Supplemental data
