# Supplementary material for: Resolution of Hypothyroidism Restores Cold-Induced Thermogenesis in Humans
Source: Thyroid. 2019 Apr 9;29(4):493–501. doi: 10.1089/thy.2018.0436 (PMC6482913; doi:10.1089/thy.2018.0436)
Supplement: Supplemental data [file Supp_Fig1.pdf]

Supplementary Data

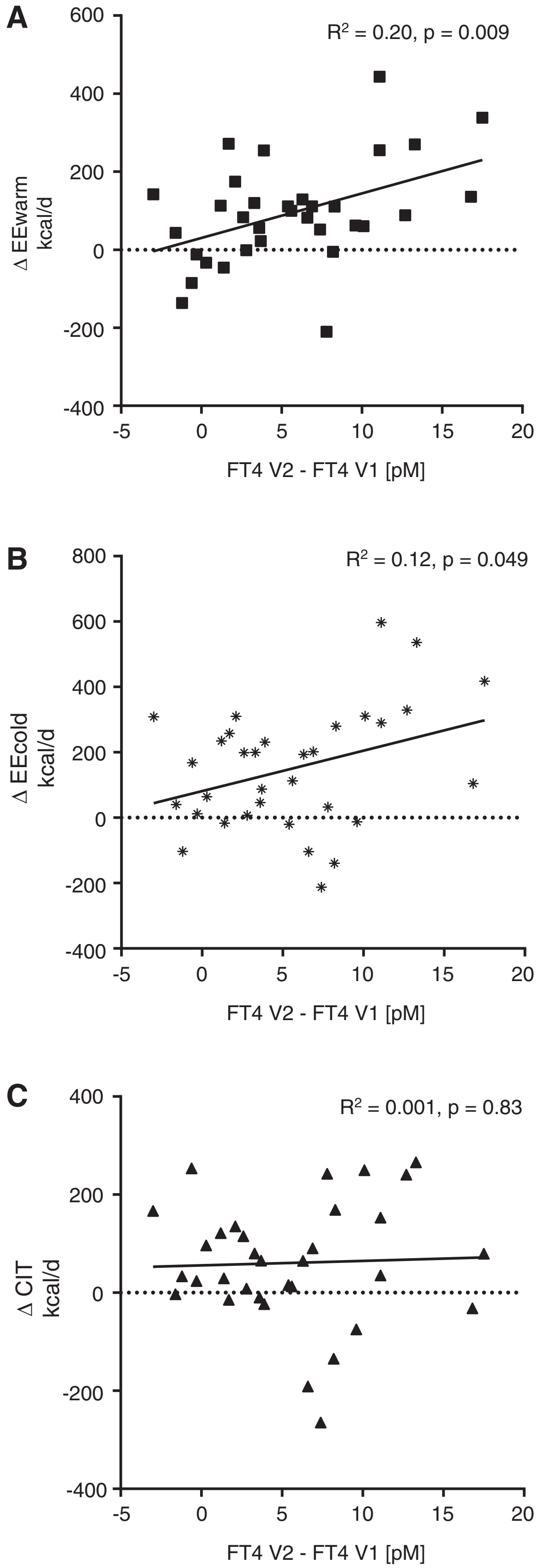

SUPPLEMENTARY FIG. S1. Relation between change in thyroid hormone status calculated as difference of free thyroxin levels (fT4) at the two visits and change in energy expenditure: (**A**) ΔEE_{warm}: $R^2 = 0.20$, $p = 0.009$; (**B**) ΔEE_{cold}: $R^2 = 0.12$, $p = 0.049$; (**C**) ΔCIT: $R^2 = 0.001$, $p = 0.83$. V1, visit 1 (hypothyroid); V2, visit 2 (euthyroid).
