# Supplementary material for: Resolution of Hypothyroidism Restores Cold-Induced Thermogenesis in Humans
Source: Thyroid. 2019 Apr 9;29(4):493–501. doi: 10.1089/thy.2018.0436 (PMC6482913; doi:10.1089/thy.2018.0436)
Supplement: Supplemental data [file Supp_Fig2.pdf]

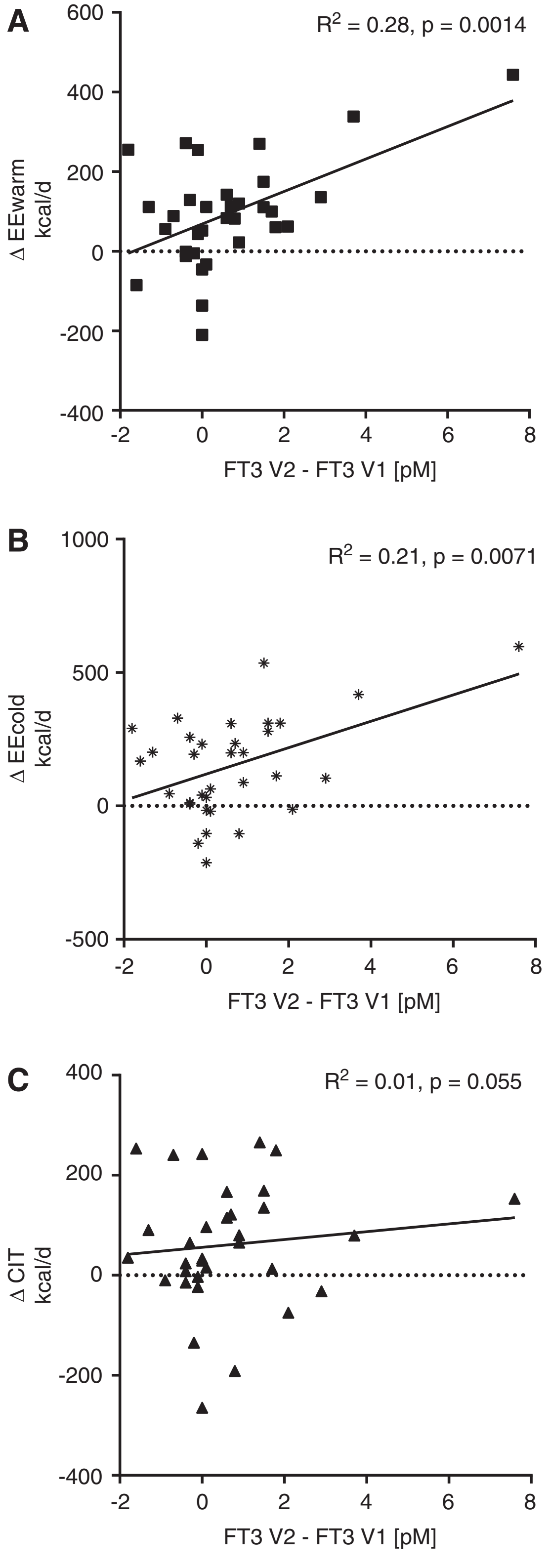

SUPPLEMENTARY FIG. S2. Relation between change in thyroid hormone status calculated as difference of free triiodothyronine levels (fT3) at the two visits and change in energy expenditure: (**A**) ΔEE_{warm}: $R^2=0.28$, $p=0.0014$; (**B**) ΔEE_{cold}: $R^2=0.21$, $p=0.0071$; (**C**) ΔCIT: $R^2=0.01$, $p=0.55$.
